# Closed Genome Sequence of Vibrio cholerae O1 El Tor Inaba Strain A1552

**DOI:** 10.1128/genomeA.00098-18

**Published:** 2018-03-01

**Authors:** Anna Allué-Guardia, Mylea Echazarreta, Sara S. K. Koenig, Karl E. Klose, Mark Eppinger

**Affiliations:** a Department of Biology, University of Texas at San Antonio, San Antonio, Texas, USA; b South Texas Center for Emerging Infectious Diseases (STCEID), University of Texas at San Antonio, San Antonio, Texas, USA

## Abstract

Vibrio cholerae is a Gram-negative waterborne human pathogen and the causative agent of cholera. Here, we present the complete genome sequence of the seventh pandemic O1 biovar El Tor Inaba strain A1552 isolated in 1992. This clinical strain has served as an important model strain for studying cholera pathogenicity traits.

## GENOME ANNOUNCEMENT

Vibrio cholerae is a waterborne pathogen that represents a public health threat affecting 3 to 5 million people worldwide (1–4). Infections are associated with the toxigenic O1 serogroup, comprised of two main biotypes, classical and El Tor, which evolved from independent lineages (5). Pathogenicity in humans is linked to two major virulence determinants, cholera toxin (CT) (6) produced by the filamentous bacteriophage CTXφ (7, 8) and the toxin-coregulated pilus (TCP) (9). When ingested, the motile bacterium propels itself toward epithelial cells of the small intestine and adheres to the apical surface, where TCP mediates colonization of the epithelial cells and CT stimulates water and electrolyte loss from the intestine, causing severe dehydration and the characteristic “rice water” diarrhea (10, 11). Cholera pathogenesis relies on the synergistic effect of a number of pathogenicity determinants, including the ToxR signaling and quorum-sensing pathways, which coordinately regulate diverse virulence genes (12, 13).

The sequenced O1 biovar El Tor Inaba strain A1552 was isolated in 1992 from a traveler from Peru (14) at the beginning of the South American cholera outbreak in the 1990s (15, 16). Total genomic DNA was extracted with the QIAamp DNA minikit (Qiagen) following the manufacturer’s instructions. To generate a high-quality closed genome, we pursued a hybrid approach using long-read and short-read sequencing on the Oxford Nanopore MinION Mk1B and Illumina MiSeq platforms, respectively. Libraries were prepared using the Nanopore ligation sequencing kit 1D (R9 version) for MinION sequencing, and a paired-end library was prepared using the Nextera XT DNA library preparation kit (Illumina) and the MiSeq V3 600-cycle reagent kit (Illumina) for Illumina sequencing. Nanopore and Illumina reads were used to perform a hybrid assembly using SPAdes (17).

Hybrid assembly yielded two circular high-coverage (125× and 112×) chromosomes (18) with genome sizes of 3,015,092 bp and 1,0703,71 bp and GC contents of 47.7% and 46.9% for chromosomes 1 and 2, respectively, in accordance with findings for other V. cholerae O1 genomes (18). The chromosomes were annotated using the NCBI Prokaryotic Genome Annotation Pipeline (PGAP) (19). A whole-genome-derived phylogeny (data not shown) identified other O1 El Tor strains as closest relatives, including the Inaba model strain N16961 (18, 20, 21).

For virulence and resistance profiling, A1552 gene and protein inventories were queried against V. cholerae virulence and resistance databases (22, 23). Strain A1552 carries genetic hallmarks of the El Tor biotype, such as the *tcp* operon, the CTXφ bacteriophage hemolysin genes, and genes associated with the ToxR regulon (24–26). *In silico* antimicrobial susceptibility testing by ResFinder (23) revealed that the smaller chromosome carries the chloramphenicol resistance gene *catB9*. This strain is also streptomycin resistant, mediated by a mutation (K88R) on the large chromosome within the 30S ribosomal subunit protein RpsL, as described previously (27, 28). The availability of the complete and closed genome will provide the blueprint for better understanding pathogenesis traits in this important prototypical O1 model strain.

### Accession number(s).

The annotated chromosomes have been deposited in GenBank under accession numbers CP025936 and CP025937.
